# Supplementary figures and images for: Aurintricarboxylic acid is a canonical disruptor of the TAZ-TEAD transcriptional complex
Source: PLoS One. 2022 Apr 13;17(4):e0266143. doi: 10.1371/journal.pone.0266143 (PMC9007350; doi:10.1371/journal.pone.0266143)

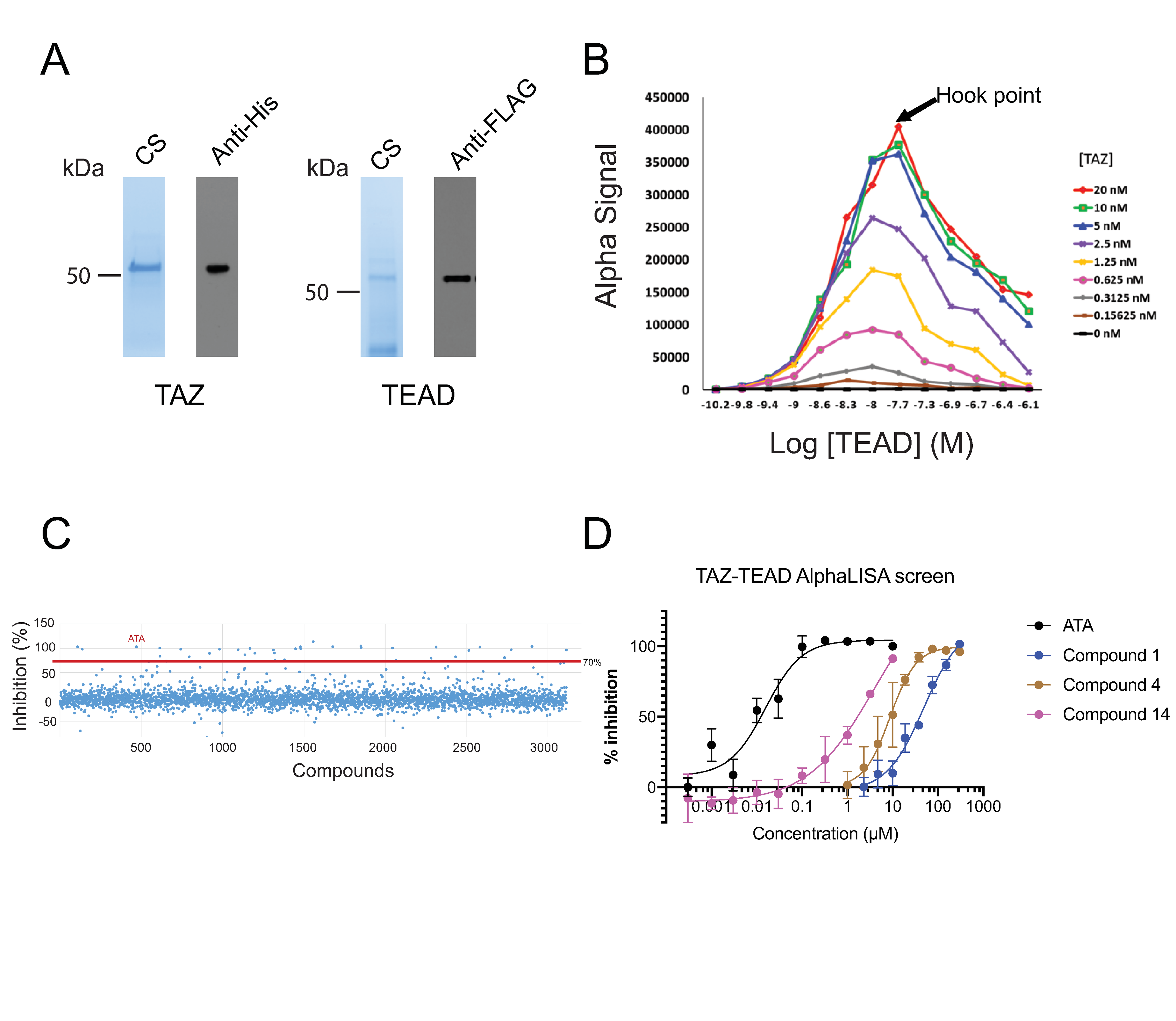

Supplement: S1 Fig — (A) Coomassie stain (CS) and western blots of full-length purified TAZ and TEAD that were used in the TAZ-TEAD AlphaLISA assay. (B) Various ratios of full-length TAZ and TEAD were used to identify the concentrations that produce the maximal alpha signal, seen at the “hook” point. (C) Distribution plot of the bioactive screen, compounds that showed greater than 70% inhibition were shortlisted as hits. (D) Dose-response curves of ATA and other ATA analogs that show inhibition in the TAZ-TEAD AlphaLISA screen. (TIF) [file pone.0266143.s001.tif]

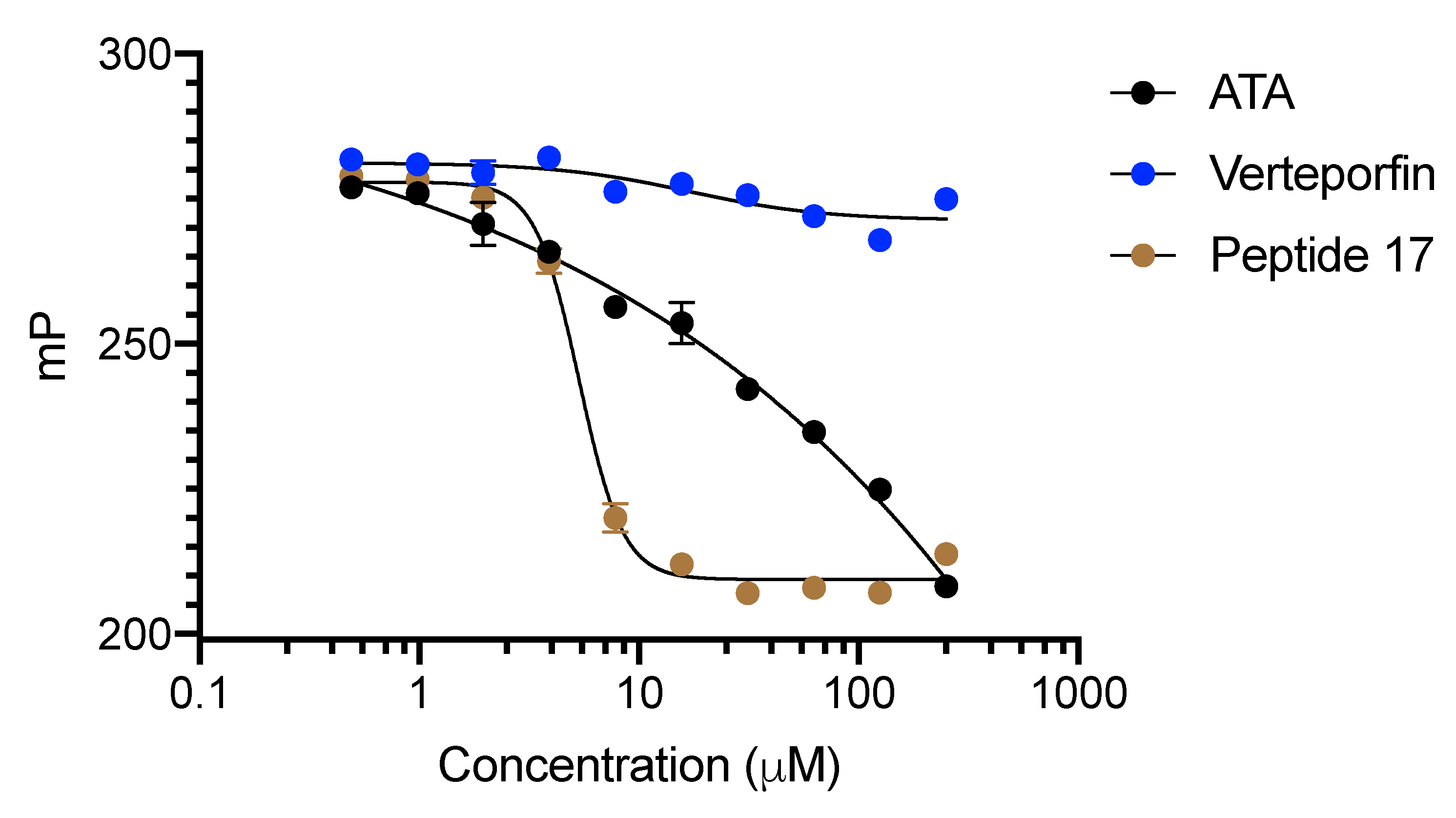

Supplement: S2 Fig — The effectiveness of ATA was compared with that of verteporfin and peptide 17. Both verteporfin and peptide 17 were identified as disruptors of the TEAD complex. Verteporfin did not display an effect in this assay. (TIF) [file pone.0266143.s002.tif]

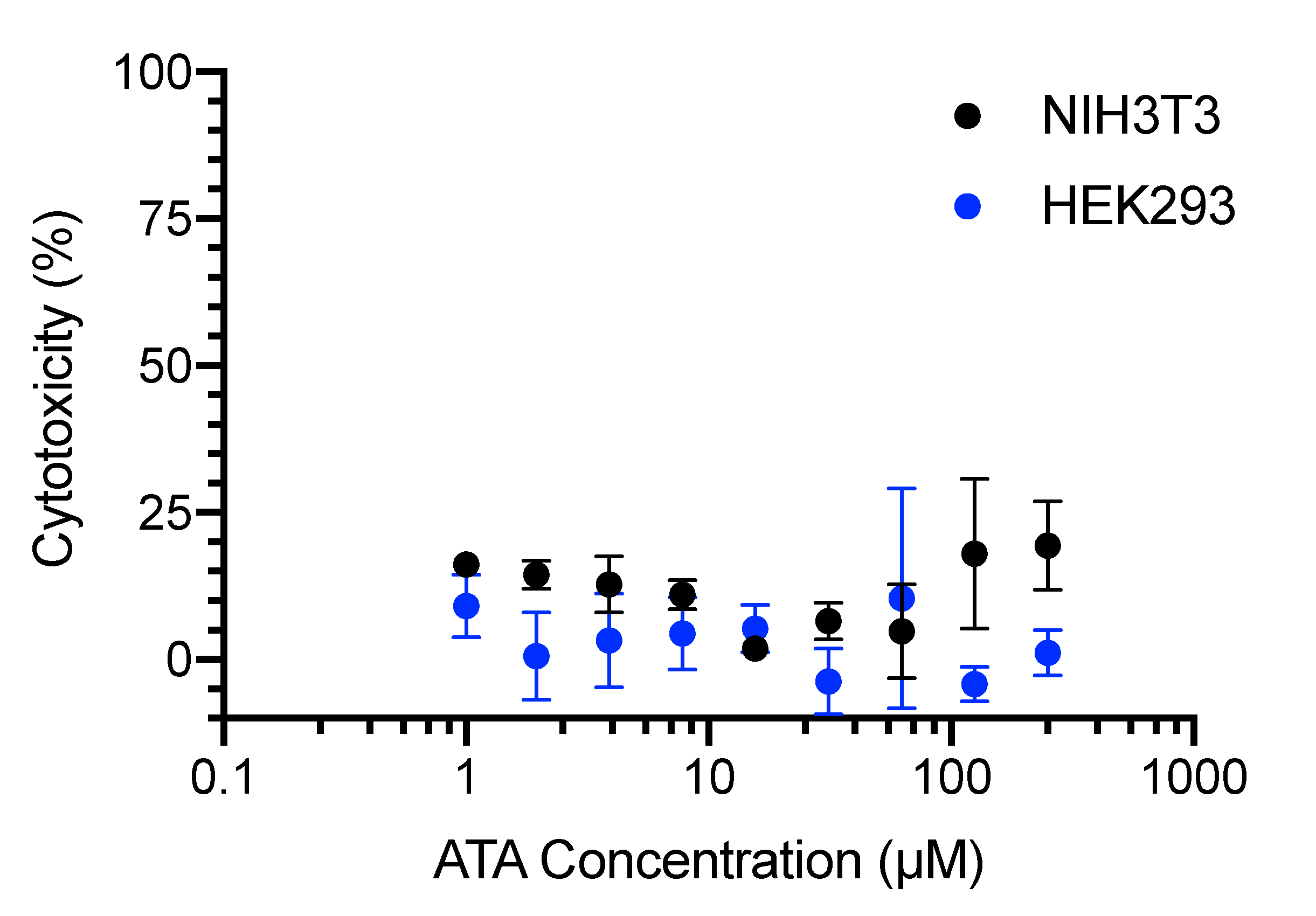

Supplement: S3 Fig — NIH3T3 and HEK293 cells were treated with the indicated concentrations of ATA and the cytotoxicity was evaluated by calculating the amount of intracellular LDH released into the media. LDH absorbance after complete cell lysis is considered as 100% cytotoxicity and the absorbance after ATA treatment was normalized accordingly. (TIF) [file pone.0266143.s003.tif]

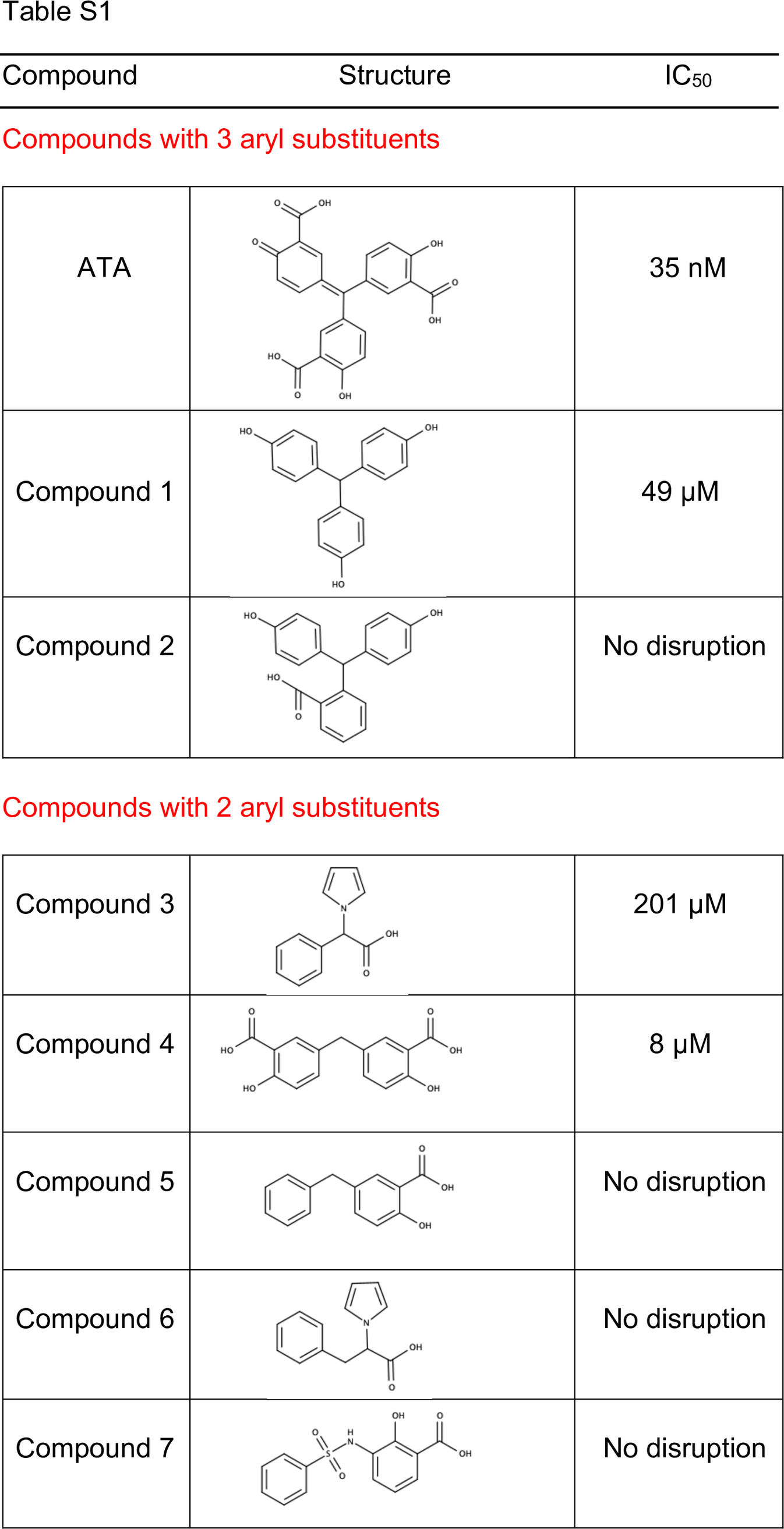

Supplement: S1 Table — (ZIP) [file pone.0266143.s004.zip › S1_Table_page 1.tif]

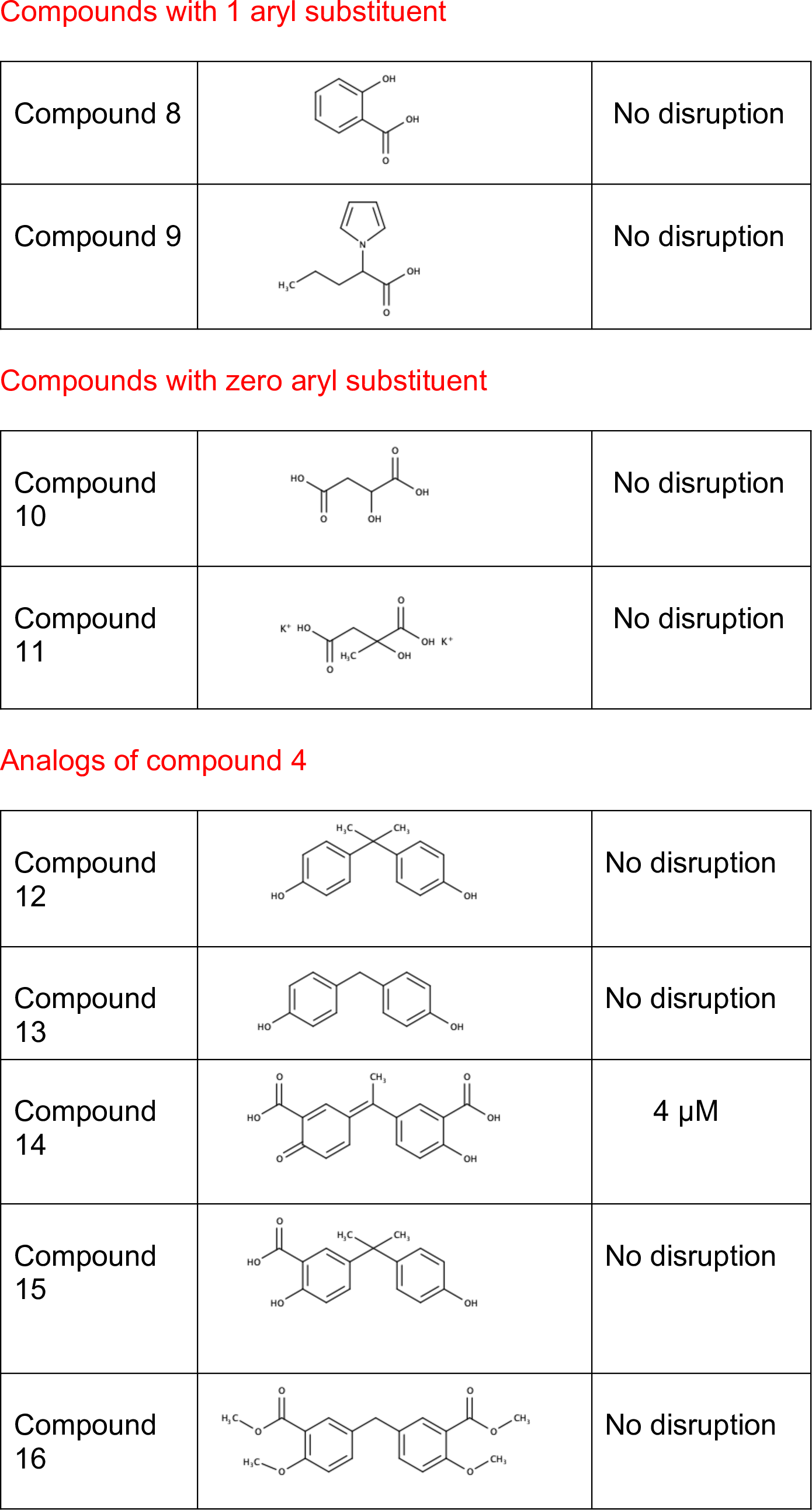

Supplement: S1 Table — (ZIP) [file pone.0266143.s004.zip › S1_Table_page 2.tif]

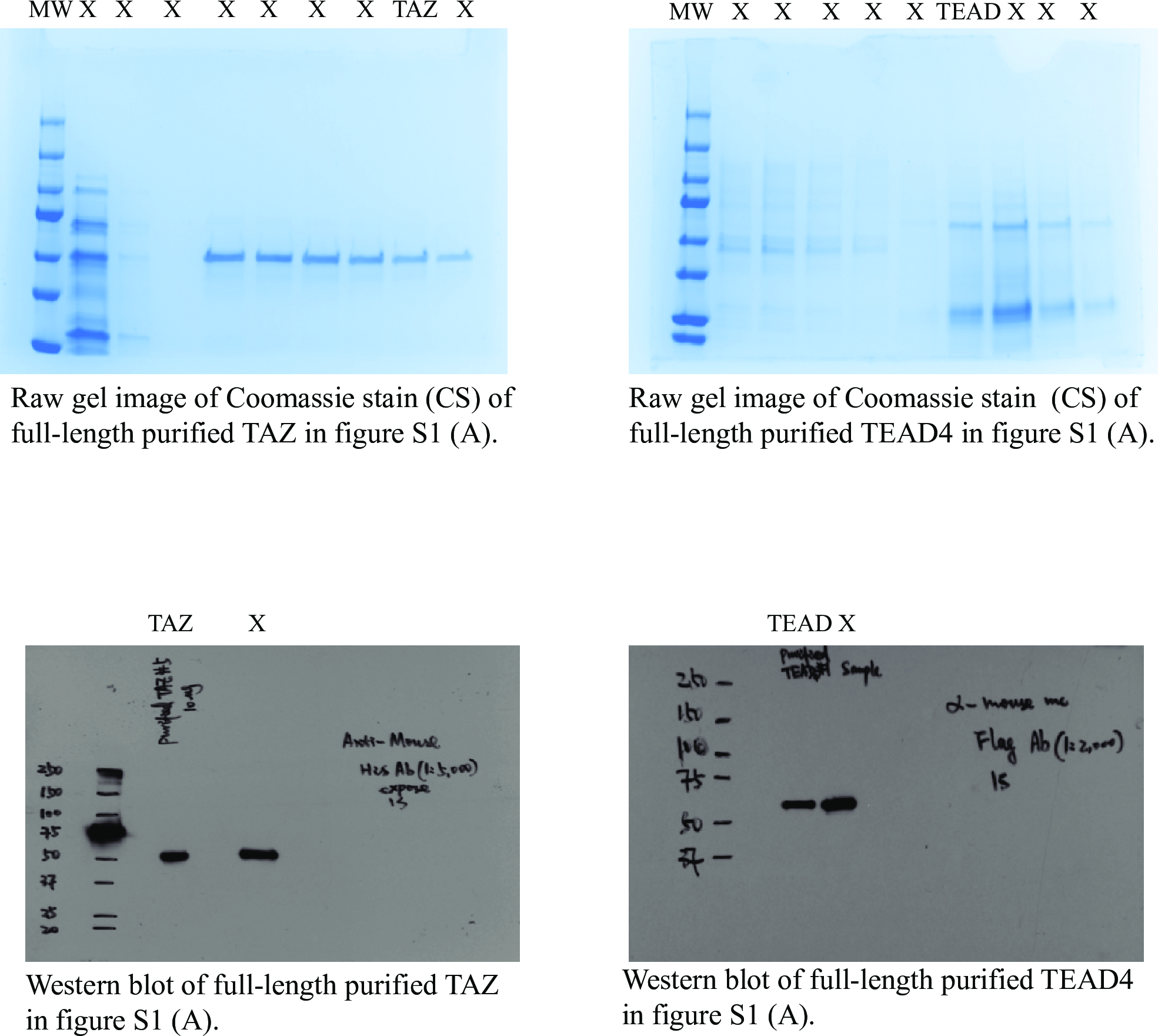

Supplement: S1 Raw images — (TIF) [file pone.0266143.s006.tif]
